# Liposomes and Extracellular Vesicles as Distinct Paths Toward Precision Glioma Treatment

**DOI:** 10.3390/ijms26146775

**Published:** 2025-07-15

**Authors:** Wiktoria Fraczek, Maciej Szmidt, Kacper Kregielewski, Marta Grodzik

**Affiliations:** 1 Department of Nanobiotechnology, Institute of Biology, Warsaw University of Life Sciences, 02-787 Warsaw, Poland; kacper_kregielewski@sggw.edu.pl; 2 Department of Morphologic Sciences, Institute of Veterinary Medicine, Warsaw University of Life Sciences, 02-787 Warsaw, Poland; maciej_szmidt@sggw.edu.pl

**Keywords:** liposomes, extracellular vesicles, glioblastoma, exosomes, drug delivery, chemotherapy, immunotherapy, gene therapy

## Abstract

Glioblastoma multiforme (GBM), the most aggressive and therapy-resistant glioma subtype, remains an urgent clinical challenge due to its invasive nature, molecular heterogeneity, and the protective constraints of the blood–brain barrier (BBB). Liposomes and extracellular vesicles (EVs) have emerged as two of the most promising nanocarrier systems capable of overcoming these limitations through improved drug delivery and cellular targeting. Their applications in glioma therapy span chemotherapy, immunotherapy, and gene therapy, each presenting distinct advantages and mechanisms of action. Liposomes offer structural flexibility, controlled release, and a well-established clinical framework, while EVs provide innate biocompatibility, low immunogenicity, and the ability to mimic natural intercellular communication. Both systems demonstrate the capacity to traverse the BBB and selectively accumulate in tumor tissue, yet they differ in scalability, cargo loading efficiency, and translational readiness. Comparative evaluation of their functions across therapeutic modalities reveals complementary strengths that may be leveraged in the development of more effective, targeted strategies for glioma treatment.

## 1. Introduction

Gliomas, the most common and aggressive primary tumors of the central nervous system (CNS), remain a challenge to clinical oncology due to their highly infiltrative growth, genetic heterogeneity, and resistance to traditional therapies [1]. The most fatal subtype of gliomas is glioblastoma multiforme (GBM), which has a median survival of approximately 15 months even with multimodal treatment that includes chemotherapy, radiotherapy, and surgical intervention [2,3]. Therapeutic failure in GBM is not only caused by resistance mechanisms, but also by the physical and structural limitations of the blood–brain barrier (BBB), which reduces the delivery of drugs administered systemically [4]. Liposomes, because of their structural adaptability and versatility, allow for precise control over pharmacokinetics and biodistribution and are one of the most well-known nanocarriers. For this reason, they have been extensively explored over the past decades for encapsulating various therapeutic agents, especially chemotherapeutics [5,6]. On the other hand, extracellular vesicles (EVs), secreted by cells as part of physiological processes, offer a biomimetic platform that can functionally replace synthetic delivery vehicles. Particularly when derived from neural or glioma-associated cells, their endogenous origin and membrane-bound ligands confer inherent biocompatibility and targeting ability [7,8,9]. Both systems’ potential has been extended beyond chemotherapy by recent developments, which demonstrated that EVs and liposomes can increase safety and efficacy [10,11]. These advancements highlight their evolving role as multifunctional platforms for precision-targeted and patient-tailored glioma treatment strategies [12].

This review aims to comprehensively compare the biological properties, delivery mechanisms, therapeutic applications, and clinical translation challenges of EVs and liposomes in the treatment of gliomas. Emphasis is placed on their role in immunotherapy, gene therapy, and chemotherapy, as well as their growing potential in the development of precision and multimodal glioma therapies. To better understand the potential of these nanocarriers, it is essential to first examine their structural foundations and functional characteristics.

## 2. Overview of Liposomes and EVs

Liposomes and EVs are two structurally related yet mechanistically distinct nanocarrier systems [13,14,15,16,17,18]. They differ in composition, biological behavior, and engineering potential due to their origins—liposomes are synthetic, EVs are endogenous [19,20,21].

This structural arrangement and origin-specific properties provide a foundation for their potential therapeutic application in gliomas [18,19,22].

### 2.1. Structural Features and Classification of Liposomes

Most liposomes are spherical nanocarriers composed of an aqueous core surrounded by one or more phospholipid bilayers. This amphiphilic structure enables the transport of a wide range of therapeutics by allowing hydrophilic drugs to be encapsulated in the aqueous core and hydrophobic compounds to be embedded within the lipid bilayer [13,16].

**Figure 1:**
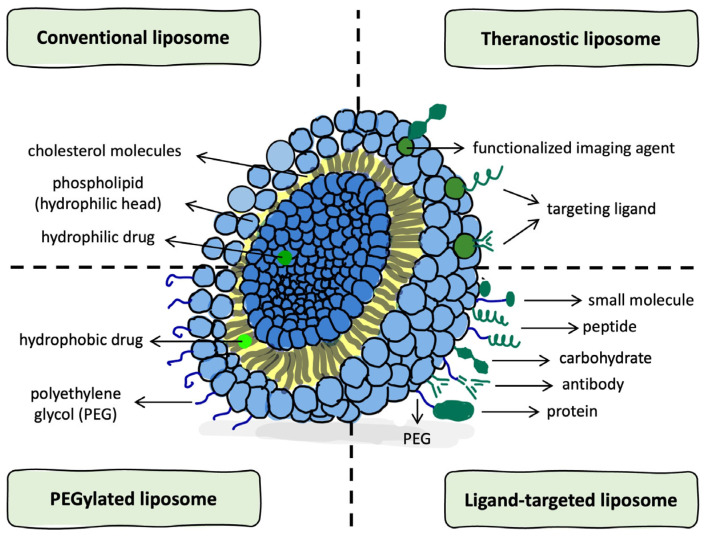
Schematic representation of liposome types and structural components. Created using the GoodNotes application (v6 1.5; GoodNotes Limited, Hong Kong, China), and adapted from Sercombe et al., [17] (Front. Pharmacol., CC BY 4.0).

As synthetic analogs of biological membranes, liposomes offer significant versatility in terms of surface modification and pharmacokinetic adjustment [17] (Figure 1).

Their in vivo behavior is influenced by various physicochemical properties, including vesicle size, surface charge, lipid composition, and the presence of functional surface modifications. Typically, liposomes range in size from 50 to 200 nanometers, while both smaller and larger structures are also utilized depending on the intended application [13,16,23].

Structurally, liposomes are classified according to size and lamellarity into small unilamellar vesicles (SUVs), large unilamellar vesicles (LUVs), multilamellar vesicles (MLVs), multivesicular vesicles (MVVs), and giant unilamellar vesicles (GUVs) [23]. These types differ not only in lamellarity and size but also in encapsulation capacity and structural resemblance to naturally occurring EVs. For instance, SUVs and LUVs are more comparable in scale and organization to exosomes, whereas MVVs share similarities with larger microvesicles [16,17,23] (Table 1).

In addition to structural classifications, liposomes can also be functionally categorized based on surface modifications and formulation strategies. Conventional liposomes rely on a passive targeting mechanism, primarily through the enhanced permeability and retention (EPR) effect, which facilitates nanoparticles to accumulate in tumor tissues through leaky vasculature and poor lymphatic drainage [16,24]. However, this approach may be limited in gliomas, where vascular abnormalities are less pronounced, leading to suboptimal drug accumulation [25]. To enhance pharmacokinetics, liposomes incorporate polyethylene glycol (PEG) chains that reduce protein adsorption and immune clearance, thereby prolonging systemic circulation [5,15].

In contrast, active targeting strategies involve functionalizing the liposomal surface with specific ligands that mediate receptor-dependent uptake by target cells. These ligands include peptides that bind to overexpressed receptors such as integrins or transferrin receptors [26,27], antibodies that enable high-affinity and selective recognition of tumor-associated antigens [27,28], and carbohydrates that exploit lectin-mediated endocytosis or glycan–receptor interactions [29,30] (Figure 1). These molecular modifications improve the selectivity and internalization of liposomes by glioma cells or brain endothelial cells, enhancing therapeutic efficacy.

Finally, theranostic liposomes integrate therapeutic agents with diagnostic imaging probes, enabling simultaneous drug delivery and real-time monitoring of treatment. Temperature-sensitive liposomes co-loaded with paclitaxel (PTX) and near-infrared II (NIR-II) fluorescent dyes (referred to as PATSL) have demonstrated the potential for in vivo image-guided glioblastoma therapy, combining chemotherapy with photothermal activation under 808 nm laser excitation [31]. Additionally, liposomes encapsulating gadolinium-based MRI contrast agents, such as IL-13-conjugated liposome–Gd-DTPA formulations, have enabled MRI-guided tracking of liposomal biodistribution across the BBB and into intracranial tumors [32].

Together, these structural and functional subtypes provide a flexible and modular platform to address key challenges in glioma therapy, including BBB penetration, tumor-specific targeting, and the delivery of multimodal treatment regimens.

### 2.2. Structural and Biogenetic Properties of EVs

EVs are lipid bilayer-enclosed particles that act as a natural mediator of intercellular communication, offering a biologically derived alternative to synthetic nanocarriers [18]. They are lipid bilayer-enclosed particles that act as mediators of intercellular communication, offering a biologically derived alternative to synthetic nanocarriers. Functionally, EVs play a central role in the horizontal transfer of bioactive molecules—such as proteins [20] and nucleic acids [18]—between different cell types, thereby modulating various physiological and pathological processes.

**Figure 2:**
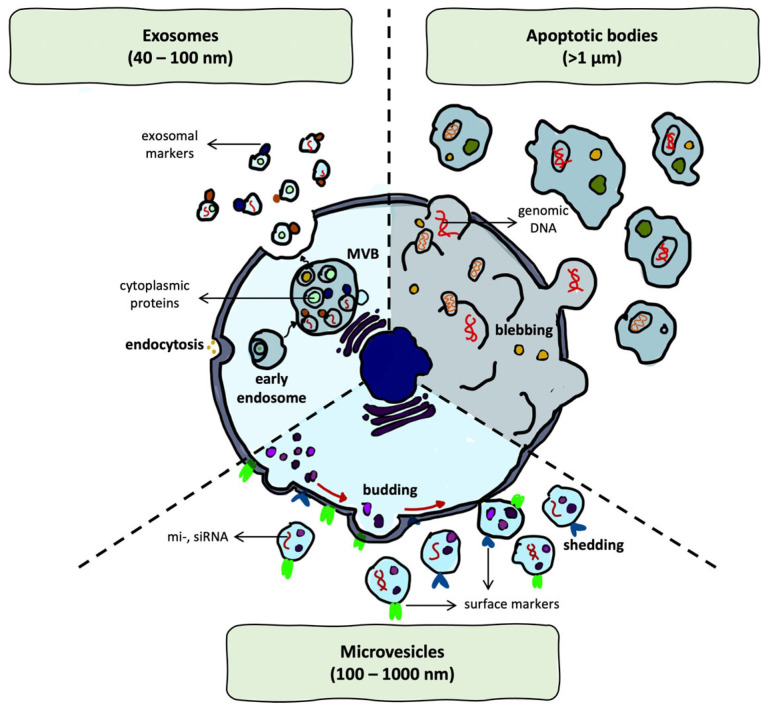
Schematic representation of extracellular vesicle biogenesis. Created using the GoodNotes application (v6 1.5; GoodNotes Limited, Hong Kong, China).

Therefore, due to their endogenous origin and biological compatibility, they are considered promising therapeutic vectors. Their functional heterogeneity arises not only from the diversity of their cargo but also from their cellular sources and distinct biogenetic pathways. Broadly, EVs are classified into three main subtypes based on size, molecular composition, and mechanisms of biogenesis: exosomes (40–100 nm), microvesicles (100–1000 nm), and apoptotic bodies (>1 µm) [18,20] (Figure 2).

The best-known and most extensively studied EVs are exosomes, which are formed via the endosomal pathway. During this process, intraluminal vesicles accumulate within multivesicular bodies (MVBs), which subsequently fuse with the plasma membrane to release their contents into the extracellular space. In contrast, microvesicles originate from the outward budding of the plasma membrane, while apoptotic bodies are released during the late stage of apoptosis as irregularly shaped vesicles containing fragmented cellular components [18,33,34].

The membrane composition of all EVs reflects both their cellular origin and their biogenetic pathway. Typical surface markers include tetraspanins such as CD9, CD63, and CD81 [35], integrins that mediate adhesion [36], and heat shock proteins, which are involved in vesicle stability and cell recognition [20,34]. These membrane components critically influence EV stability, cellular uptake, and targeting.

Internally, EVs may carry cytoplasmic proteins, messenger RNAs (mRNAs), microRNAs (miRNAs) [37], and genomic DNA (gDNA) [37,38]. Importantly, cargo loading is thought to occur via both passive diffusion and active, regulated sorting mechanisms [39].

Thus, the structural heterogeneity and endogenous origin of EVs underpin their functional versatility as nanocarriers in biomedical applications.

## 3. Applications of EVs and Liposomes in Glioma Therapy

**Figure 3:**
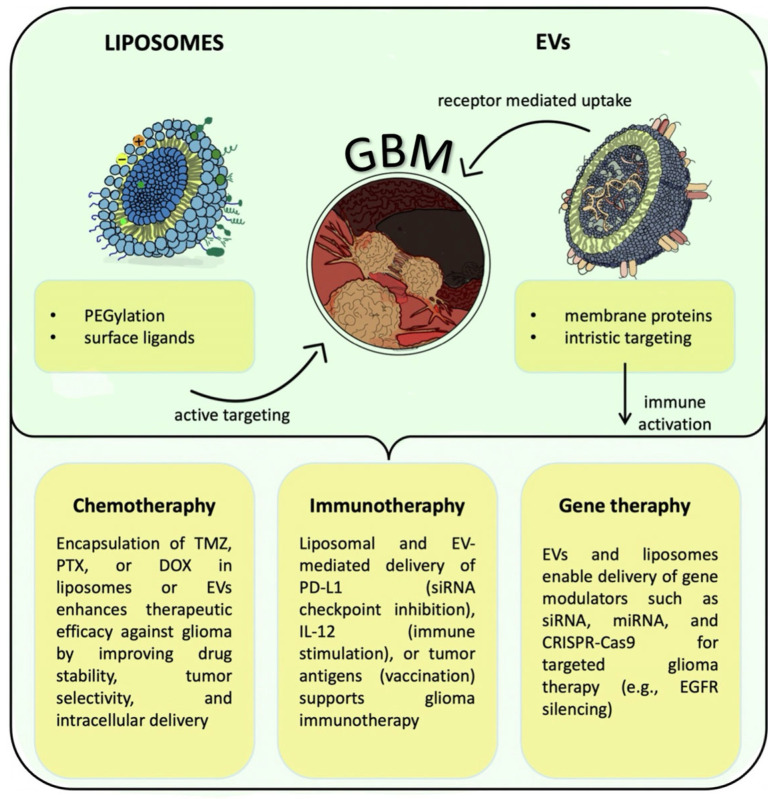
Schematic overview of liposome- and EV-based drug delivery strategies in glioma therapy. Created using the GoodNotes application (v6 1.5; GoodNotes Limited, Hong Kong, China).

Both EVs and liposomes are gaining increasing relevance in various aspects of glioma treatment owing to their versatile and promising therapeutic potential [17,18]. Targeted delivery of immunomodulators, nucleic acids, or chemotherapeutic agents is possible due to their ability to encapsulate a wide range of active substances [5,9,12,20]. This approach could enhance therapeutic efficacy while minimizing systemic toxicity [10]. Nanocarriers provide unique benefits and mechanisms of action, facilitating their incorporation into glioma treatment strategies under development [40,41] (Figure 3).

### 3.1. Chemotherapy

Chemotherapy remains a cornerstone in the treatment of glioblastoma multiforme, but numerous pharmacological and biological obstacles have consistently and significantly reduced its effectiveness [42,43,44]. This paradox is exemplified by the current chemotherapeutic gold standard, Temozolomide (TMZ) [2,44,45]. Systemic toxicity [2,44,45], low bioavailability [45], and most importantly, resistance mechanisms mediated by O6-methylguanine-DNA methyltransferase (MGMT) [44] often limit the clinical potential of TMZ. By repairing the DNA damage that TMZ is thought to cause, MGMT acts as a molecular shield inside tumor cells, reducing the therapeutic efficacy of TMZ and causing adverse clinical outcomes [44].

Similar pharmacological limitations apply to chemotherapeutics such as Doxorubicin (DOX) and PTX, which, despite encouraging results in preclinical glioma models, have shown limited success in clinical GBM trials. Consequently, drug delivery systems based on nanocarriers, particularly liposomes, have gained attention as a means to overcome these challenges [5,16,46]. Functionalized and dual-targeting nanocarriers are currently under investigation to further optimize delivery and tumor specificity [47].

Among the most promising strategies to overcome these limitations is the use of liposomal formulations, which enable improved pharmacokinetics and targeted delivery, especially for chemotherapeutics with low solubility, rapid systemic clearance, or high toxicity [5,16]. Although TMZ and PTX differ in their molecular structure, mechanisms of action, and approved indications, their free-form administration suffers from similar limitations, including poor tumor accumulation, rapid systemic clearance, low aqueous solubility, and high off-target toxicity [5,16]. However, these agents can be encapsulated in a liposomal bilayer to improve their therapeutic profile [5,16,48,49].

A notable example is PEGylated liposomal TMZ formulated as an LUV structure, which exhibits extended systemic circulation and improved brain tumor accumulation. In U87 glioma cells, this formulation decreased the IC_50_ of TMZ from ~77 µg/mL to 4 µg/mL, induced apoptosis in ~19% of cells, and significantly increased cellular uptake [48]. Comparable findings were reported in C6 and U251 glioma cell lines, where PEGylated liposomal TMZ exhibited approximately 1.6-fold higher cytotoxicity compared to the free drug formulation [49].

The therapeutic advantages of liposomal encapsulation have also been validated in vivo, where targeted formulations improved drug accumulation in tumor tissue and significantly extended survival in glioma-bearing animals [46,50,51]. In a preclinical study, Lin et al. utilized convection-enhanced delivery (CED) to administer PEGylated liposomal TMZ (8.7 mg/mL) directly into the brains of U87G glioma-bearing nude mice. While a single 5 μL dose had a negligible impact on survival, repeated administration 7 days later extended median survival to over 70 days, without observable systemic toxicity or local tissue damage [50]. A comparable therapeutic outcome was observed using PEGylated liposomes composed of 1,2-distearoyl-sn-glycero-3-phosphocholine (DSPC) and cholesterol, delivering TMZ at a concentration of 1.7 mg/mL via convection-enhanced delivery. This formulation also demonstrated significant survival benefits in glioma-bearing animals, confirming the robustness of the CED-based liposomal approach [51].

Also, PTX, a well-established anti-proliferative agent, has demonstrated limited clinical success in glioblastoma therapy due to its poor aqueous solubility and significant neurotoxicity. However, its therapeutic potential has been revitalized through encapsulation in liposomal formulations—MLV form and PEGylated liposomes in LUV configurations, which enhance drug solubility, stability, and tumor delivery [52,53]. In an orthotopic glioblastoma mouse model, menthol-modified liposomal PTX led to a ~66% reduction in tumor volume and extended median survival from 22 to 36 days compared to the free drug. Notably, this formulation also minimized systemic toxicity, underscoring the therapeutic advantages of liposomal delivery [52]. Likewise, PTX-loaded liposomes modified with a multifunctional R8–RGD tandem peptide exhibited enhanced glioma accumulation and achieved the longest survival among all treatment groups in C6 glioma-bearing mice. These effects were accompanied by clear apoptotic activity and no observable systemic toxicity, highlighting the potential of peptide-functionalized liposomes in glioma therapy [53].

Although the encapsulation method may vary depending on the physicochemical properties of each drug (requiring different lipid compositions or loading techniques), the overarching advantages remain consistent [5,16,17]. Liposomal delivery improves pharmacokinetics, facilitates tumor targeting, and enables the reformulation of existing chemotherapeutics into more viable treatment options for GBM [5,16,17]. The successful application of this strategy to both TMZ and PTX underscores the versatility of liposomal carriers in addressing the diverse challenges of chemotherapeutic delivery in glioma therapy [5,31]. To build on these benefits, recent efforts have aimed at improving tumor specificity through active targeting.

To further enhance tumor specificity and maximize therapeutic efficacy, liposomal formulations have been engineered with surface ligands—such as angiopep-2 or CD133 antibodies conjugated to PEGylated liposomes in an LUV structure—that facilitate active targeting [54]. In orthotopic glioma models, liposomes functionalized with both angiopep-2 and anti-CD133 antibodies demonstrated significantly higher tumor accumulation, a 2.4-fold increase in apoptosis, and improved treatment outcomes compared to non-targeted controls [54]. These targeting strategies improve not only cellular uptake efficiency but also penetration into the tumor microenvironment, particularly in hard-to-reach niches such as glioma stem-like cell reservoirs.

Beyond monotherapy, liposomal platforms support combinatorial treatments, including multimodal regimens. Co-administration of liposomal TMZ with bromodomain inhibitors has yielded synergistic anti-tumor effects in preclinical glioma models, underscoring the versatility of liposomes as modular carriers. This synergy stems from the ability of bromodomain inhibitors—particularly those targeting BRD4—to suppress oncogenic transcriptional programs and DNA repair pathways that are otherwise activated in response to TMZ-induced damage, thereby enhancing glioma cell sensitivity to alkylating agents. Beyond their role in chemosensitization, BRD4-targeting bromodomain inhibitors have also been shown to enhance anti-tumor immunity by promoting antigen presentation and reducing T cell exhaustion [55,56].

While liposomes offer a well-established and customizable delivery platform, EVs represent a biologically derived alternative that may overcome some of the fundamental limitations of synthetic carriers. By mimicking natural cell-to-cell communication, EVs enable intrinsically targeted delivery that requires no artificial surface modification. Their membranes are naturally enriched with adhesion molecules and surface proteins that facilitate selective uptake by glioma cells, thereby minimizing off-target accumulation and enhancing therapeutic precision [7,8,9,34].

In the context of GBM chemotherapy, exosomes—the smallest and most studied EV subtype—have gained increasing attention as particularly promising carriers. The therapeutic promise of exosome-based delivery systems has been well demonstrated in vitro. In glioma cell cultures, PTX-loaded exosomes derived from U87 cells significantly reduced cell viability to around 60% within 48 h—clearly outperforming free PTX in the same conditions [57]. Building on this, folic acid-conjugated EVs co-delivering TMZ and quercetin (TMZ–Qct–FA–Exo) further enhanced cytotoxic effects, inhibiting GBM cell proliferation more effectively than either drug alone [58]. Yet, exosomes do not merely serve as passive carriers; those secreted by TMZ glioma cells have been shown to transmit resistance phenotypes by delivering miR-1238 to native recipient cells, thereby reducing their apoptotic response and diminishing drug sensitivity [59].

These in vitro results provide a compelling rationale for further evaluating the in vivo pharmacological performance of exosome-based formulations. Their nanoscale dimensions and endogenous membrane composition support prolonged systemic circulation, facilitate deeper tumor tissue penetration, and promote efficient intracellular drug delivery [19,23,42,43]. These attributes translate into tangible therapeutic benefits, as demonstrated in preclinical models. In orthotopic GBM-bearing mice, paclitaxel-loaded exosomes achieved approximately 2.3-fold higher intratumoral drug concentrations and induced a ~45% reduction in tumor volume compared to free PTX [57]. Similarly, systemic administration of folic acid-conjugated exosomes co-delivering TMZ and quercetin reduced tumor burden by ~50% and extended median survival from 28 to 45 days in glioma-bearing rats, relative to untreated controls [58].

Though exosomes dominate the EV landscape, other subtypes such as microvesicles and apoptotic bodies are also being explored for drug delivery [59]. Microvesicles offer a larger cargo capacity and can be loaded with chemotherapeutic agents to improve their stability and uptake. PTX-loaded microvesicles derived from autologous prostate cancer cells (LNCaP and PC-3 cell lines) significantly enhanced the cytotoxicity of paclitaxel, reducing cell viability by approximately 40–50% in comparison to free PTX. However, despite these promising results, the larger size of microvesicles presents challenges for efficient delivery [59].

Apoptotic bodies, while capable of carrying substantial drug loads, are similarly limited by their size but may hold promise in enhancing drug delivery due to their unique biological roles. Studies have shown that apoptotic bodies can be loaded with various bioactive molecules and drugs, significantly improving their therapeutic potential. These bodies are naturally capable of fusing with cell membranes, facilitating the release of their payload inside target cells. Despite these promising capabilities, their larger size can limit their ability to efficiently penetrate tissues, which poses challenges for broader clinical applications [60].

However, it is essential to note that neither microvesicles nor apoptotic bodies have been specifically investigated in the context of GBM. Although both exhibit promising potential in other cancer models, their application in glioma therapy remains largely unexplored. As the therapeutic landscape for GBM continues to evolve, exosomes are emerging as a transformative platform, offering a naturally targeted and multifunctional approach that not only complements but, in some cases, surpasses the capabilities of traditional liposomal systems.

Together, these findings illustrate the complementary strengths of liposomal and EV-based delivery platforms in glioma chemotherapy, as summarized in Table 2.

### 3.2. Immunotherapy

In addition to chemotherapy, EVs and liposomes have gained increasing attention as platforms for modulating the immune response in GBM. Immunotherapy has emerged as a compelling strategy in the treatment of GBM, offering the potential to shift the focus from traditional cytotoxic strategies to immune-based interventions. However, the clinical success of immunotherapy in GBM has been limited by its profoundly immunosuppressive tumor microenvironment (TME). This immune evasion is driven by poor infiltration of cytotoxic T lymphocytes and a high abundance of regulatory immune cells, including tumor-associated macrophages (TAMs), regulatory T cells (Tregs), and myeloid-derived suppressor cells (MDSCs) [3,67,68]. Moreover, the overexpression of checkpoint molecules such as PD-L1 further suppresses immune responses, ultimately leading to T cell exhaustion and tumor progression [67,68].

Meta-analysis by Zeng et al. evaluating the efficacy and safety of anti-PD-1/PD-L1 therapy in glioma patients reported that, while the therapy was generally well tolerated, it did not significantly prolong survival [69]. The pooled estimate for median overall survival was 8.85 months, with a 1-year overall survival rate of 43%. The median progression-free survival was 3.72 months, with a 1-year progression-free survival rate of 15%. These findings highlight the limited clinical benefit of PD-1/PD-L1 inhibitors in glioma and reinforce the urgency of exploring complementary or alternative approaches to enhance therapeutic efficacy.

One promising approach to overcoming these immunotherapeutic limitations involves the use of liposomal nanocarriers. A study by Pourmasoumi et al. demonstrated that albumin–liposome nanoparticles, designed to deliver siPD-L1, significantly improved the therapeutic outcomes compared to free siRNA. In in vivo glioma models, when administered intracranially to GL261 tumor-bearing mice, liposomal siPD-L1 resulted in approximately 2.3-fold greater accumulation within tumor tissue, compared to free siRNA, resulting in a ~45% reduction in tumor volume. Moreover, a marked increase in tumor-infiltrating CD8^+^ T cells indicated functional reactivation of local anti-tumor immunity. These results suggest that liposomal systems could effectively deliver immune checkpoint inhibitors, significantly enhancing therapeutic efficacy in GBM [58].

An important innovation in liposomal immunotherapy involves the co-delivery of immune checkpoint inhibitors alongside tumor-associated antigens and immunostimulatory adjuvants. Although this strategy has primarily been explored in non-glioma tumor models, it provides a compelling conceptual framework that may inform future combinatorial approaches in glioblastoma treatment [70].

In parallel to these synthetic strategies, EVs are being explored as a distinct class of immunotherapeutic vectors, offering complex immunological functionalities derived from their cellular origin. These nanoscale vesicles carry immune-relevant proteins, lipids, and nucleic acids within a membrane that reflects the surface markers of the parent cell, enabling communication with immune components in a highly selective manner [71].

Dendritic cell-derived exosomes (Dexosomes) have garnered significant attention as acellular vaccines in cancer immunotherapy due to their natural expression of MHC molecules and co-stimulatory ligands [37]. These vesicles can effectively stimulate cytotoxic T cell responses by mimicking professional antigen-presenting cells. Building on this concept, Bu et al. demonstrated that dendritic cell-derived exosomes loaded with tumor-associated peptides could induce a robust anti-tumor immune response in glioma patients. In their study, exosomes were enriched with MAGE-1 antigen and immune-stimulatory molecules such as MHC-I and HSP70. Upon incubation with these engineered vesicles, dendritic cells activated CD8^+^ cytotoxic T lymphocytes (CTLs), which exhibited selective cytotoxicity against GBM cells, while sparing control lymphoblasts. The resulting immune response contributed to the targeted rejection of glioma cells, illustrating the therapeutic potential of exosome-based immunotherapies in GBM treatment [72].

Moreover, exosomes have recently emerged as promising vehicles for the delivery of immune checkpoint inhibitors, further enhancing anti-tumor immune responses. Notably, exosome-mediated delivery of PD-L1-targeting siRNA has been shown to effectively silence PD-L1 expression on tumor cells, thereby restoring T cell activity. Similar to liposomes, exosomes can facilitate the targeted inhibition of this critical immune checkpoint. Notably, their inherent biological properties (such as enhanced cellular uptake and immune compatibility) may confer additional advantages over synthetic systems. In a study by Himes et al., exosomal siPD-L1 delivery led to a marked reduction in PD-L1 levels, accompanied by increased infiltration of CD8^+^ cytotoxic T lymphocytes into glioblastoma tissue [73]. This dual effect—simultaneous checkpoint inhibition and immune activation—demonstrates the therapeutic potential of exosome-based strategies for overcoming immune resistance in GBM.

Beyond exosomes, alternative EV subtypes, including microvesicles and apoptotic bodies, are also under investigation for their immunomodulatory potential in glioma. Microvesicles have been shown to modulate the immune landscape by engaging tumor-associated macrophages and myeloid-derived suppressor cells, promoting PD-L1 expression and reinforcing immunosuppression [74,75].

Moreover, apoptotic bodies released by dying GBM cells also play a dual role. On one hand, they have been implicated in promoting tumor progression through the horizontal transfer of oncogenic components such as RNA-binding proteins involved in alternative splicing [76]. On the other hand, their biological features (including membrane-bound phosphatidylserine and enhanced uptake by phagocytic immune cells) make them promising carriers for immunomodulatory cargos. While the use of engineered apoptotic bodies in GBM remains largely conceptual, their capacity to selectively interact with dendritic cells and macrophages could be leveraged to deliver checkpoint inhibitors or tumor antigens across the BBB [77,78].

Together, these examples highlight the distinct yet complementary mechanisms by which EVs and liposomes can enhance the precision and effectiveness of immunotherapy in GBM (Table 3).

### 3.3. Gene Therapy

Gene therapy has recently emerged as a transformative strategy in glioma treatment, shifting the therapeutic paradigm from direct cytotoxicity to the genetic reprogramming of tumor and immune cells [11]. Rather than eliminating cancer cells outright, this approach aims to correct the molecular drivers of glioma by silencing oncogenes, reactivating tumor suppressors, or modulating the immunosuppressive tumor microenvironment [20,80]. However, the clinical implementation of gene therapy in glioblastoma multiforme has been hindered by several obstacles, particularly the instability of nucleic acids in systemic circulation and limited cellular uptake [11,20].

To address these challenges, nanocarriers have been increasingly adapted to protect and deliver gene-based therapeutics with enhanced specificity and reduced toxicity. Among these, liposomes have shown promise, as they can encapsulate a variety of genetic materials, including siRNAs [81] and mRNAs [82]. One compelling example of liposome-based gene therapy involves the targeted silencing of STAT3, a key transcription factor implicated in glioma progression and immune evasion. In a study by Linder et al., hybrid lipopolyplex nanoparticles composed of low-molecular-weight polyethylenimine and DPPC lipids were engineered to encapsulate STAT3-specific siRNA. These ~130 nm particles, exhibiting near-neutral zeta potential, were administered intracranially into immunocompetent mice bearing syngeneic Tu2449 gliomas. The treatment led to a 50–65% reduction in STAT3 expression in vitro and significantly inhibited tumor growth in vivo, ultimately prolonging median survival compared to untreated controls [81].

Beyond STAT3, liposomal RNA interference strategies have also been applied to target other tumor-intrinsic drivers in glioma. Resnier et al. employed lipid nanocapsules to deliver siRNA against EGFR into U87MG glioma cells, resulting in approximately 63% knockdown of EGFR expression and a 38% reduction in cell proliferation in vitro. In a clinically relevant model, liposomal delivery of MGMT-specific siRNA via the LipoTrust™ EX Oligo system (Hokkaido System Science Co., Ltd., Sapporo, Japan) effectively reversed TMZ resistance in glioma-initiating cells [83]. The siRNA-loaded liposomes were administered both intratumorally in a subcutaneous xenograft model and via osmotic pump infusion in an orthotopic intracranial glioma model. This dual-mode delivery led to robust MGMT knockdown and significantly enhanced TMZ sensitivity, resulting in marked tumor growth inhibition in vivo, without inducing neurotoxicity [84].

Moreover, lipid nanoparticle (LNP)-mediated mRNA delivery has recently emerged as a powerful immunotherapeutic strategy. In a study by Hamouda et al., intratumoral administration of LNPs encoding IL-21, IL-7, and 4-1BBL promoted strong CD8^+^ T cell infiltration and enhanced expression of granzyme B and Interferon gamma. This treatment led to complete tumor regression in approximately 86% of glioma-bearing mice and established long-term immunological memory, particularly when combined with PD-1 checkpoint blockade [82].

While overlap exists—especially in cases where gene-based tools (e.g., siRNA or mRNA) influence immune signaling—the classification depends primarily on therapeutic intent. If the goal is to directly modulate immune activation (e.g., via checkpoint blockade or cytokine expression), the intervention is generally considered immunotherapy. In contrast, if the objective is to regulate gene expression within tumor or immune cells to alter their intrinsic behavior, the strategy is classified as gene therapy [85,86]. This conceptual distinction justifies treating gene therapy as a separate category in this section, even when immunomodulatory outcomes are involved (Table 4).

Building upon the success of RNA-based approaches, researchers are now turning to CRISPR–Cas9 genome editing, enabled by advanced liposomal platforms for more durable and precise interventions in glioma.

More recently, liposomal CRISPR–Cas9 systems have emerged as effective tools for genome editing in glioblastoma. A notable innovation is the polymer-locking fusogenic liposome (“Plofsome”), which encapsulates CRISPR–Cas9 ribonucleoprotein (RNP) complexes and remains inert in circulation but becomes fusogenic upon exposure to elevated reactive oxygen species in the tumor microenvironment. In orthotopic glioma models, Zhao et al. demonstrated that these liposomes successfully crossed the BBB and delivered both Cas9 RNP and siRNA directly to tumor cells. Targeted editing of the MDK oncogene resulted in ~60% gene editing efficiency, accompanied by a ~70% reduction in MGMT expression and a ~50% inhibition of tumor growth, while sparing healthy brain tissue. These findings underscore the potential of CRISPR–Cas9-loaded liposomes to simultaneously reverse chemoresistance and suppress glioma progression [11,89].

Like synthetic platforms, EVs have also been engineered to deliver CRISPR–Cas9 complexes for site-specific genome editing in glioma. Their endogenous membrane composition, low immunogenicity, and natural tropism for brain tissue make them particularly attractive vehicles for gene editing. In a recent study, Li et al. developed dual-modified exosomes functionalized with Angiopep-2 and TAT peptides to encapsulate Cas9–sgRNA complexes targeting the *glutathione synthetase (GSS)* gene [90]. Following intratumoral injection in orthotopic glioblastoma models, these EVs achieved a 67.2% reduction in GSS expression, significantly enhanced radiosensitivity, and suppressed tumor progression—all without detectable off-target effects or neurotoxicity. These results highlight the potential of EV-mediated CRISPR delivery for precise and durable genomic reprogramming in GBM.

Beyond their engineered applications, EVs possess intrinsic therapeutic and pathological potential owing to their endogenous cargo and cellular origin. EVs derived from glioma stem-like cells (GSCs), MSCs, or immune cells naturally carry nucleic acids and proteins that participate in intercellular communication, immune modulation, and tumor progression [91]. Their membrane composition—rich in adhesion molecules, tetraspanins, and integrins—not only facilitates selective uptake by glioma cells but also confers protection from immune clearance [18].

A compelling example is provided by Bao et al., who demonstrated that exosomes derived from GSCs are highly enriched in miR-155-5p, showing more than a threefold increase compared to exosomes from non-malignant glial cells. These exosomes were found to target the tumor suppressor *ACOT12* in recipient glioma cells, resulting in significant upregulation of mesenchymal markers such as *TWIST2* and vimentin, alongside enhanced cell migration and invasion in vitro. In an in vivo xenograft model, administration of miR-155-5p antagomirs led to a ~50–55% reduction in tumor volume and mass over a 28-day period, without adverse effects on animal health. These findings underscore the pathogenic potential of endogenous EVs and their active contribution to glioma progression [88].

While exosomes have received the greatest attention in EV-based gene therapy, other subtypes, such as microvesicles and apoptotic bodies, are increasingly being explored in other cancer models as alternative gene delivery platforms. Owing to their larger size and distinct biogenesis, microvesicles can accommodate complex cargos such as Cas9 RNPs and long RNAs and have been successfully engineered for tumor-targeted CRISPR/Cas9 delivery in hepatocellular and breast cancer models [92]. Likewise, apoptotic bodies have been repurposed as gene delivery tools by exploiting their natural uptake via efferocytosis and their capacity to carry high molecular weight DNA and protein complexes [78]. Although these strategies have not yet been validated in glioma models, their favorable properties—including natural biocompatibility, high cargo load, and immune evasion—suggest they may serve as valuable platforms for future gene-based interventions in GBM.

Recent advances in gene therapy for glioma have demonstrated that both liposomal and EV-based systems can effectively deliver nucleic acid cargos, including siRNA, mRNA, and CRISPR–Cas9 constructs. Preclinical studies have shown improved target gene knockdown, tumor suppression, and survival outcomes using these platforms. While exosomes are the most widely studied, early-stage investigations suggest that microvesicles and apoptotic bodies may also serve as viable gene carriers. A comparative summary of liposomal and EV-based strategies in gene therapy for glioma is presented in Table 5.

## 4. Challenges and Future Directions

The emergence of EVs and liposomes as drug delivery systems in glioma therapy represents a pivotal shift in translational nanomedicine. Both platforms have demonstrated strong potential in preclinical models [5,11,96]. As these technologies move closer to clinical application, it becomes increasingly clear that their success will depend not only on continued innovation but also on overcoming distinct challenges. Understanding these obstacles and the complementary strengths of each platform is crucial for advancing clinically viable nanocarrier-based therapies [97,98].

### 4.1. The Complexity of Crossing the Brain’s Defenses

Among the most persistent challenges in glioma therapy is the presence of the BBB, a tightly regulated endothelial interface that restricts the passage of most therapeutics into the central nervous system [4]. More than a structural filter, the BBB is an active and adaptive physiological defense, influenced by both systemic factors and local changes within the tumor microenvironment [94]. For systemically administered therapies, this barrier remains a major obstacle, limiting both drug exposure and therapeutic efficacy.

To overcome the restrictive nature of the BBB, liposomes require surface modifications that enable active transport into the brain. Due to their physicochemical properties, such as hydrophilic surfaces and relatively large size, liposomes are typically unable to cross the BBB via passive diffusion [95]. Instead, they must be actively guided across the barrier using molecular ligands that engage receptor-mediated transport pathways. Common strategies involve conjugation with targeting moieties such as transferrin [26], the arginine–glycine–aspartic (RGD) motif [27], or angiopep-2 [54], all of which mimic endogenous mechanisms to facilitate transcytosis [19].

These modifications have been shown to significantly enhance cellular uptake and tissue specificity, particularly in glioblastoma models [27]. However, they also introduce considerable complexity and variability in formulation and reproducibility, which remain key challenges in the clinical translation of targeted liposomal systems [64].

Mechanistically, the traversal of the BBB by such nanocarriers relies primarily on receptor-mediated transcytosis (RMT), and to a lesser extent on adsorptive-mediated transcytosis (AMT). RMT is triggered by specific ligand–receptor interactions, leading to endocytosis on the luminal side of endothelial cells, intracellular trafficking, and exocytosis at the abluminal membrane [26,27,64]. While AMT can facilitate BBB crossing, it is generally less selective and efficient. The effectiveness of both mechanisms depends on nanoparticle properties such as size, surface charge, and ligand density, as well as on the physiological state of the BBB. In glioma, the barrier may become partially compromised, resulting in heterogeneous permeability and altered transcytosis activity [4,19].

While particle size is one of the most widely reported parameters influencing nanocarrier passage across the BBB, there is no universally accepted threshold that guarantees penetration. Nanoparticles within the 50–150 nm range are typically considered optimal for BBB translocation, especially when assisted by active transport mechanisms. However, size is only one of many contributing factors; surface charge, lipid composition, PEGylation, ligand functionalization, and the physiological state of the BBB all modulate transport efficacy. For instance, PEGylated liposomes of ~55 nm have demonstrated enhanced brain accumulation compared to larger counterparts (~120–200 nm) in healthy models [16,99], whereas glioma-associated BBB disruption can allow passage of larger constructs.

Beyond their size, liposomes offer pharmacokinetic advantages stemming from their bilayer structure, which allows for controlled and sustained release of encapsulated drugs. This not only protects sensitive therapeutic payloads from enzymatic degradation but also extends drug exposure at the tumor site [46]. Nonetheless, many preclinical liposomal strategies that successfully cross the BBB do so with the aid of adjunctive techniques, such as focused ultrasound, osmotic opening, or convection-enhanced delivery, which may limit their clinical applicability [100,101].

Although liposomes remain one of the most structurally versatile and clinically validated nanocarriers [6], their efficient delivery to the brain typically requires synthetic modifications such as PEGylation and ligand conjugation. These strategies, while effective, introduce challenges including immune recognition, endosomal escape, and batch-to-batch variability [102]. Moreover, reliance on external interventions to facilitate BBB crossing adds complexity and limits their scalability [100,101]. As a result, growing interest has turned toward alternative delivery platforms with innate biological compatibility and minimal need for engineering. Among these, EVs have emerged as promising candidates for CNS drug delivery.

Because of their origin, EVs appear naturally adapted to navigate the complex defenses of the brain. Instead of relying on disruptive methods or synthetic modifications, EVs harness endogenous transport mechanisms to cross the BBB with minimal perturbation [7,8,60,103]. This intrinsic compatibility with physiological barriers has positioned them as an increasingly attractive platform for CNS-targeted therapies.

Multiple studies have demonstrated that EVs derived from immune cells, neural stem cells, or glioma cells can not only traverse the BBB but also selectively accumulate in glioma tissue [9,103]. This targeting ability is attributed to specific surface moieties retained from the parent cells, including tetraspanins (CD9, CD63, CD81), integrins (e.g., αvβ3, α6β4), and phosphatidylserine, which interact with overexpressed receptors on the glioma endothelium and tumor cells. Notably, EVs from GBM or neural progenitor sources often display ligands such as CD44, L1CAM, or EGFRvIII, enabling homotypic recognition and enhanced tissue specificity [10,94].

These membrane features, inherited from the donor cells, provide EVs with a molecular “fingerprint” that facilitates both passive and active targeting within the brain. This level of selective tropism remains challenging to replicate with synthetic carriers [7,8,103].

Mechanistically, EVs utilize multiple internalization routes, including receptor-mediated transcytosis and various forms of endocytosis—such as clathrin-, caveolae-, and macropinocytosis-based uptake [104]. While their small size (40–150 nm) places them within the optimal range for BBB penetration, it is their composite structure and molecular signature that ultimately governs their delivery efficiency and selectivity.

Because of their biological evolution, EVs seem to be naturally adapted to negotiating the intricate defense mechanisms of the brain. Their subtle and biologically informed delivery pathway is made possible by their minimally disruptive ability to cross the BBB [7,8,94,105].

As a result, although both systems show promise, EVs may offer a more physiologically harmonious route across the BBB, while liposomes continue to evolve through increasingly sophisticated engineering strategies to match this potential.

### 4.2. Cargo Capacity vs. Cargo Compatibility

In GBM therapy, the clinical success of a nanocarrier depends not only on tumor accumulation but also on how well the vehicle accommodates therapeutic payloads and releases them in a controlled and biologically compatible manner. This includes protection of fragile molecules, evasion of premature degradation, and efficient intracellular delivery [11,89]. In this context, liposomes and EVs offer complementary strengths and weaknesses: liposomes provide structural tunability and high loading capacity [13,66], while EVs exhibit cargo-preserving properties and cell-specific uptake profiles due to their biological origin [9,30].

Liposomes allow for precise engineering of their lipid bilayers, enabling integration of pH-sensitive, enzyme-cleavable, or temperature-responsive moieties that promote stimulus-triggered drug release in the GBM microenvironment [13,16,64,106]. Their internal compartments can accommodate various small molecules and nucleic acids, and their surface can be decorated with ligands to improve targeting. Nevertheless, efficient intracellular delivery of macromolecular cargo remains limited. Following endocytosis, liposomes often become sequestered within lysosomes, leading to cargo degradation or incomplete release [84,106].

Although PEGylation can prolong systemic circulation and reduce immune clearance, it also introduces new challenges. Repeated dosing of PEGylated formulations may induce the accelerated blood clearance (ABC) phenomenon, complicating long-term treatment [107,108]. While some PEGylated liposomal drugs, such as Doxil (Janssen Products, LP, Horsham, PA, USA), have demonstrated clinical efficacy in non-CNS cancers, this success has not yet been fully replicated in GBM therapy [5].

EVs, in contrast, offer a delivery mechanism shaped by biological function. Their membrane composition, enriched in lipids such as phosphatidylserine and sphingomyelin, along with surface proteins including tetraspanins and integrins, supports natural interactions with recipient cells, particularly those of neural origin [9]. This configuration aids both cargo stability and cytoplasmic delivery. Unlike many synthetic vectors, EVs can enter cells through receptor-mediated uptake or membrane fusion and may facilitate endosomal escape via back-fusion processes or lipid-driven destabilization of the endosomal membrane [109,110,111].

However, EVs also face major limitations, particularly in the context of exogenous cargo loading. While intrinsic packaging mechanisms enable efficient incorporation of endogenous molecules, external therapeutics are far more difficult to introduce. Passive loading methods often result in low drug concentrations, while active strategies, such as electroporation, sonication, or saponin-mediated permeabilization, can disrupt vesicle integrity, lower targeting fidelity, or induce aggregation [42,104]. These interventions risk compromising the very biological features that make EVs attractive in GBM therapy.

To address these limitations, emerging strategies such as donor-cell engineering offer promising alternatives. Genetic modification of glioma- or neural progenitor-derived donor cells using tools like CRISPR/Cas9 can enhance the homogeneity, targeting capacity, and cargo-loading efficiency of EVs intended for GBM applications [9]. A seminal example of this approach is the study by Alvarez-Erviti et al. [94], in which dendritic cells were engineered to express a neuron-targeting peptide (RVG) fused to the exosomal membrane protein Lamp2b. The resulting exosomes, loaded with siRNA, were able to cross the BBB and selectively silence gene expression in neuronal tissue in vivo, demonstrating the translational potential of donor-cell engineering in central nervous system disorders. Click-chemistry has also been explored to enable specific ligand conjugation to the EV surface, potentially improving glioma-specific uptake. Most notably, strain-promoted azide–alkyne cycloaddition (SPAAC) allows for catalyst-free, bioorthogonal attachment of targeting moieties—such as folic acid or glioma-homing peptides—to EV membranes, enhancing selective internalization by tumor cells in vivo [103].

Membrane permeabilization via agents such as saponins has been shown to improve drug encapsulation efficiency without fully disrupting vesicle structure [42,57,103,112]. Additionally, EV–liposome hybrid platforms offer a modular solution that combines the GBM tropism of EVs with the drug release control of liposomal systems—helping to bridge the gap between biological compatibility and therapeutic capacity [42,57,103,112].

Despite these advancements, EVs still present challenges in terms of reproducibility and quality control. Unlike liposomes, whose composition can be tightly regulated during synthesis, EV populations are inherently heterogeneous in size (30–200 nm), protein content, and RNA profile—even when derived from a single glioma cell source [9,106,113]. This intrinsic variability complicates standardization and remains a major regulatory hurdle [9,109].

Ultimately, the comparison between EVs and liposomes in GBM therapy is not about identifying a universally superior platform, but about strategically aligning their physicochemical and biological properties with the demands of specific therapeutic goals (Table 6).

Liposomes offer scalable manufacturing, consistent drug loading, and a customizable release profile, making them particularly suited for small-molecule delivery and combinatorial therapies [13,109]. By contrast, EVs, though technically more challenging to manipulate and standardize, provide unparalleled biocompatibility, cell-specific tropism, and efficient delivery of fragile biologics [114,115]. As GBM therapies increasingly target intracellular mechanisms, modulate immune responses, or employ gene editing, the selection of an optimal delivery system must align both with the drug’s properties and the biological constraints of the tumor microenvironment [12,15,63].

### 4.3. The Manufacturing Divide

The successful translation of nanoparticle-based drug delivery systems from preclinical models to clinical application hinges not only on therapeutic efficacy but also on the feasibility of scalable, reproducible, and regulatory-compliant manufacturing. In this context, the contrast between liposomes and EVs is particularly instructive, underscoring the divide between a clinically established platform and one that is still emerging from early-stage development [5,17,114].

Liposomes have been extensively studied and industrially optimized for over four decades. Several liposomal formulations—including Doxil^®^ (doxorubicin; Janssen Pharmaceuticals/Johnson & Johnson, Titusville, NJ, USA), Myocet^®^ (non-PEGylated doxorubicin; Sopherion Therapeutics, Princeton, NJ, USA), Onivyde^®^ (irinotecan liposome injection; Ipsen Biopharmaceuticals, Cambridge, MA, USA), and AmBisome^®^ (liposomal amphotericin B; Gilead Sciences, Inc., Foster City, CA, USA)—have received regulatory approval for the treatment of various cancers and fungal infections. Although none are currently approved specifically for GBM, their success in other indications underscores the translational feasibility of liposomal nanocarriers [5,16].

Unlike EVs, liposomes benefit from well-established production protocols and scalable technologies. These systems are traditionally produced via methods such as thin-film hydration, ethanol injection, reverse-phase evaporation, extrusion, and microfluidic mixing, but these approaches face significant challenges in scalability [13]. One of the most promising scalable methods for liposome production is supercritical fluid (SCF) technology, which offers enhanced control over liposome size and uniformity, as well as better reproducibility on a large scale [66]. This high degree of tunability enables batch-to-batch reproducibility and integration with Good Manufacturing Practice (GMP) standards, significantly facilitating regulatory approval and commercial translation [5].

EVs, in contrast, represent a biologically sophisticated yet technologically immature class of nanocarriers. Their isolation from conditioned media or biofluids is inherently complex. Techniques such as differential ultracentrifugation, tangential flow filtration, size-exclusion chromatography, and immunoaffinity capture each introduce trade-offs in yield, purity, scalability, and vesicle integrity [5]. Moreover, defining product identity, developing potency assays, and ensuring dose consistency remain difficult due to the inherent heterogeneity of the EV population, even when derived from a single donor source [112,113]. These limitations complicate the establishment of regulatory pathways comparable to those developed for synthetic nanocarriers and hinder the development of reproducible manufacturing protocols [112].

To improve standardization, the International Society for Extracellular Vesicles (ISEV) has published the MISEV2018 guidelines, which define minimal criteria for EV characterization, including markers for identity and purity, quantification methods, and reporting standards [116]. Although the rigor of preclinical research has increased as a result, the application of these guidelines in GMP-compliant manufacturing remains limited. To date, only a handful of academic and commercial groups have initiated early-phase clinical studies using EVs produced under partially or fully GMP-compliant conditions [112].

Recent efforts to overcome these limitations have focused on the development of closed, scalable production systems. Bioreactor-based cell expansion platforms, such as hollow-fiber and stirred-tank systems, have improved the yield of EVs while minimizing batch-to-batch variability. Advances in tangential flow filtration and automated size-exclusion chromatography have enhanced purification consistency and throughput [117]. Emerging technologies such as microfluidics, designer donor cell lines, and modular EV–liposome hybrid platforms offer promising routes to overcome these barriers and improve clinical results [114]. Nonetheless, critical challenges remain in the development of real-time quality control analytics, robust potency assays, and long-term storage solutions, all of which are essential for regulatory acceptance [117].

Ultimately, the prevailing distinction between EVs and liposomes lies more in infrastructure than in inherent technological capability.

While liposomes continue to dominate the field of therapeutic nanomedicine, EV-based platforms are likely to remain confined to early-phase research until supportive manufacturing and regulatory frameworks are fully established [5,16,17,114,118].

Despite these advancements, the control of EV surface composition and targeting capacity remains a significant limitation. Unlike synthetic liposomes, whose membrane structure can be precisely engineered and replicated across batches, EVs inherit their surface moieties—including adhesion proteins, glycoproteins, and lipids—from the donor cells, which introduces biological variability [117].

Batch-to-batch heterogeneity is especially pronounced when EVs are produced under varying cell culture conditions or from heterogeneous donor populations [118]. While metabolic labeling, CRISPR-based donor cell engineering, and click-chemistry surface modifications are promising solutions to modulate EV surfaces in a controlled and reproducible way, these strategies are still under development and not yet standardized for large-scale or clinical-grade production [65,116,118]. As such, ensuring consistency in EV targeting remains an active area of investigation, and regulatory–compliant quality control frameworks must be further refined to enable broader clinical translation.

## 5. Conclusions

This study examined the growing interest in the use of liposomes and EVs as nanocarriers in GBM treatment, focusing on their distinct advantages, therapeutic applications, and translational challenges. In fields such as immunotherapy, gene therapy, and chemotherapy, both platforms show promise by enhancing targeting specificity, reducing systemic toxicity, and improving drug delivery across the BBB. Liposomes are excellent candidates for traditional drug delivery as well as multimodal approaches because of their clinical maturity, scalable production, and adjustable design. On the other hand, EVs provide immunological compatibility and cell targeting mechanisms that have evolved biologically, making them attractive vectors for immunomodulatory and nucleic acid-based treatments.

Both systems have limitations despite their potential. While EVs continue to face challenges with standardization, drug loading efficiency, and large-scale manufacturing, liposomes encounter problems with immune clearance and endosomal trapping. These difficulties highlight the necessity of more research, not only to improve individual platforms but also to better integrate them into treatment paradigms that are clinically relevant.

Rather than proposing a universal solution, this analysis supports a more nuanced conclusion. The choice between liposomes and EVs must be guided by the nature of the therapeutic agent, the treatment objective, and the specific biological barriers posed by GBM. In doing so, nanocarrier-based delivery systems may contribute to the development of more personalized, effective, and less invasive interventions for one of neuro-oncology’s most challenging diseases.

## Figures and Tables

**Table 1:** Structural and functional categorization of liposomes relevant to biomedical applications.

Type	Size Range	Lamellarity	Encapsulation Capacity	Biological Analogy	References
Small Unilamellar Vesicle (SUV)	20–100 nm	Single bilayer	Low (limited aqueous volume)	Structurally and dimensionally comparable to exosomes	[16,23]
Large Unilamellar Vesicle (LUV)	100–1000 nm	Single bilayer	Moderate to high	Overlap in size with larger exosomes and early endosomes	[13,17,23]
Multilamellar Vesicle (MLV)	>500 nm	Multiple concentric bilayers	Low (limited aqueous space)	May resemble multilamellar EVs or complex apoptotic bodies	[16,23]
Multivesicular Vesicle (MVV)	>500 nm	Outer bilayer enclosing internal vesicles	High (dual aqueous compartments)	Comparable to multivesicular bodies or large EV subtypes	[17,23]
Giant Unilamellar Vesicle (GUV)	>1 µm	Single bilayer	Variable (suitable for large macromolecules)	Used as membrane models; less common in vivo	[23]

**Table 2:** Comparative overview of liposomal and EV-based chemotherapy delivery.

Feature	Liposomes	Extracellular Vesicles	References
Drug Encapsulation Efficiency	High encapsulation efficiency is possible, but highly dependent on the preparation method, lipid composition, and vesicle size	Moderate and less tunable; limited by parent cell type and passive loading approaches, size, etc.	Liposomes: [13,48,51] EVs: [58,61]
Stability in Circulation	Inherently unstable; improved by PEGylation, but remains a formulation challenge	Stable in biofluids, but stability can vary depending on isolation methods and donor cell variability	Liposomes: [5,13,16,51] EVs: [8,9,54]
Tumor Targeting	Requires the addition of targeting ligands (e.g., transferrin, angiopep)	Naturally enriched surface proteins aid glioma cell uptake	Liposomes: [26,27,54] EVs: [10,35,62,63]
Therapeutic Efficacy	Effective tumor delivery with proper formulation; relies on EPR effect and surface design	Often show superior penetration and retention in glioma models, especially in dense tumor tissue	Liposomes: [5,12,48,52,54,64] EVs: [10,56,57,58]
Immunogenicity	Potentially immunogenic, especially upon repeated administration	Low immunogenicity; naturally derived, generally well tolerated	Liposomes: [13,17] EVs: [10,57,63]
Scalability and Manufacturing	Industrial processes exist, but standard methods are not fully scalable; SCF is emerging	Lacks scalable methods; isolation and reproducibility remain major hurdles	Liposomes: [3,16] EVs: [65,66]

Note: EVs refer primarily to exosomes unless otherwise specified.

**Table 3:** Summary of liposomal and EV-based immunotherapy strategies in glioma.

Strategy	Liposomes	Extracellular Vesicles	References
Checkpoint Inhibition	Encapsulated PD-L1 siRNA or anti-PD-1 antibodies (engineered)	Engineered EVs loaded with PD-L1 siRNA	Liposomes: [8,27] EVs: [1,2,7,19,38,72]
Cancer Vaccines	Tumor-associated peptides + CpG co-loaded liposomes	Dexosomes enriched with MHC I/II and glioma-associated antigens	Liposomes: [70] EVs: [37,72]
Cytokine Delivery	IL-12-loaded liposomes improve T cell activation and anti-tumor immunity	EVs carrying IL-12 mRNA promote localized cytokine expression and immune activation in GBM	Liposomes: [79] EVs: [74,80]
Macrophage Reprogramming	Experimental delivery of immunomodulators (rarely used)	Glioma-derived EVs carrying IL-6/miR-155-3p promote M2 polarization via STAT3 activation	Liposomes: [27] EVs: [61,72,77]
Antigen Presentation/Priming	Requires synthetic adjuvants	Apoptotic bodies and Dexosomes present native tumor antigens	Liposomes: [70] EVs: [38,72,77]

Abbreviations: PD-L1, Programmed death-ligand 1; PD-1, Programmed cell death protein 1; siRNA, Small interfering RNA; CpG, Cytosine–phosphate–guanine oligonucleotide; IL-12, Interleukin-12; EVs, Extracellular vesicles; MHC, Major histocompatibility complex; STAT3, Signal transducer and activator of transcription 3; IL-6, Interleukin 6.

**Table 4:** Distinction between RNA-based immunotherapy and gene therapy applications in glioma.

Therapeutic Tool	Immunotherapy Use	Gene Therapy Use	Reference
siRNA (e.g., PD-L1)	Restores T cell activation by silencing immune checkpoints	Silences immune-suppressive genes, enhancing anti-tumor response	[39,87]
miRNA (e.g., miR-155)	Polarizes macrophages toward the M1 phenotype and boosts cytotoxic immune activity	Reprograms tumor-associated macrophages and modifies the glioma-supportive stroma	[61,88]
mRNA (e.g., IL-12)	Enables localized expression of immune-stimulatory cytokines	Introduces tumor-suppressing cytokines and therapeutic proteins through transient expression	[63,75,82]
CRISPR-Cas9	-	Enables genome editing of oncogenes and resistance-related genes like MGMT	[84,87,89]

Abbreviations: siRNA, Small interfering RNA; miRNA, MicroRNA; mRNA, Messenger RNA; CRISPR-Cas9, Clustered Regularly Interspaced Short Palindromic Repeats with associated Cas9 endonuclease; PD-L1, Programmed death-ligand 1; MGMT, O6-methylguanine-DNA methyltransferase.

**Table 5:** Comparative applications of liposomes and EVs in gene therapy for glioma.

Feature	Liposomes	Extracellular Vesicles	References
siRNA Delivery	Liposomal formulations carrying siRNA (e.g., PD-L1) inhibit glioma; require PEGylation or targeting moieties	EVs naturally internalized by glioma cells deliver siRNA via fusion or endocytosis	Liposomes: [83,84,89] EVs: [93,94]
miRNA Delivery	Liposomal miR-124 or miR-7 modulate STAT3/MGMT pathways, sensitizing glioma to chemotherapy	EVs loaded with miR-155 polarize TAMs (M1); miR-124 EVs inhibit glioma progression	Liposomes: [55,84] EVs: [61,88,95]
mRNA Delivery	Liposomes encapsulating IL-12 mRNA or mRNA vaccines trigger local immune activation	Engineered EVs deliver immune-modulating mRNA, enhancing T cell response in GBM	Liposomes: [82] EVs: [39,80]
Gene Editing	Liposomal CRISPR–Cas9 systems enable efficient knock-out of PD-L1 or MGMT	EVs co-delivering Cas9 protein demonstrate enhanced uptake and editing efficiency in glioma cells	Liposomes: [11,89] EVs: [90,92]

Abbreviations: PD-L1, Programmed death-ligand 1; STAT3, Signal transducer and activator of transcription 3; MGMT, O6-methylguanine-DNA methyltransferase; TAMs, Tumor-associated macrophages; IL-12, Interleukin-12; Cas9, CRISPR-associated protein 9; GBM, Glioblastoma multiforme.

**Table 6:** Comparative features of liposomes and extracellular vesicles in glioma therapy.

Feature	Liposomes	Extracellular Vesicles	References
Drug Encapsulation	High encapsulation efficiency; tunable by lipid composition, vesicle size, and preparation method	Moderate efficiency; dependent on parental cell type and passive loading techniques	Liposomes: [13,16,31,53] EVs: [20,57,58,78]
BBB Penetration	Requires surface modification (e.g., Angiopep-2) to cross the BBB	Crosses BBB via endogenous receptor-mediated transcytosis mechanisms	Liposomes: [5,16,26,27,66] EVs: [9,20,60,94,103]
Tumor Targeting	Achieved by attaching ligands (e.g., transferrin, folate, antibodies) to the surface	Inherent targeting potential due to membrane proteins and cell-of-origin tropism	Liposomes: [26,27,28,54] EVs: [9,35,36,94,106]
Endosomal Escape	Limited; many liposomes are degraded in endosomes unless modified with pH-sensitive components	EVs demonstrate improved cytoplasmic delivery due to their natural membrane composition	Liposomes: [16,84,106] EVs: [88,104,109,110,111]
Gene Delivery	Liposomes deliver siRNA, mRNA, and CRISPR-Cas9 components via encapsulation and surface engineering	EVs naturally carry and transfer miRNAs, siRNAs, and CRISPR-Cas9 machinery	Liposomes: [11,82,83,84,86,89] EVs: [18,61,90,94]
Clinical Scalability	Scalable using established pharmaceutical manufacturing processes; proven in other cancer types	Scalability remains challenging due to heterogeneity and a lack of standardized production methods	Liposomes: [5,16,17,64] EVs: [65,112,113,114]
Innovative Hybrids	Liposome–EV hybrid vesicles under development; aim to improve delivery and targeting	Hybrid EV mimetic platforms show enhanced uptake and controlled drug release	Liposomes: [12,64] EVs: [12,35]

Abbreviations: BBB, Blood–brain barrier; EVs, Extracellular vesicles; siRNA, Small interfering RNA; miRNA, MicroRNA; CRISPR-Cas9, Clustered Regularly Interspaced Short Palindromic Repeats associated protein 9; Angiopep-2, Peptide ligand facilitating BBB transcytosis.

## Data Availability

The data presented in this study are available upon request to the corresponding author.

## Abbreviations

The following abbreviations are used in this manuscript:

CNS	Central Nervous System
GBM	Glioblastoma multiforme
BBB	Blood–Brain Barrier
EVs	Extracellular Vesicles
SUVs	Small Unilamellar Vesicles
LUVs	Large Unilamellar Vesicles
MLVs	Multilamellar Vesicles
MVVs	Multivesicular Vesicles
GUVs	Giant Unilamellar Vesicles
EPR	Enhanced Permeability and Retention
PEG	Polyethylene glycol
MVBs	Multivesicular bodies
PTX	Paclitaxel
NIR-II	Near-infrared II
mRNA	Messenger ribonucleic acid
miRNA	Micro-ribonucleic acid
gDNA	Genomic DNA
TMZ	Temozolomide
MGMT	O-6-methylguanine-DNA methyltransferase
CED	convection-enhanced delivery
DSPC	1,2-distearoyl-sn-glycero-3-phosphocholine
DOX	Doxorubicin
SCF	Supercritical fluid
TME	Tumor microenvironment
TAMs	Tumor-associated macrophages regulatory
Tregs	Regulatory T cells
MDSCs	Myeloid-derived suppressor cells
siRNA	small interfering ribonucleic acid
PD-L1	Programmed death-ligand 1
CpG	Cytosine–phosphate–guanine oligonucleotide
CTL	Cytotoxic T lymphocyte
Dexosomes	Dendritic cell-derived exosomes
MHC	Major histocompatibility complex
IL-2	Interleukin-2
IL-12	Interleukin-12
PD-1	Programmed cell death protein 1
STAT3	Signal transducer and activator of transcription 3
Il-6	Interleukin-6
IL-21	Interleukin-21
IL-7	Interleukin-7
LPN	Lipid nanoparticle
GSC	Glioma stem-like cell
CRISPR-Cas9	Clustered Regularly Interspaced Short Palindromic Repeats associated protein 9
EGFR	Epidermal growth factor receptor
RMT	Receptor-mediated transcytosis
AMT	Adsorptive-mediated transcytosis
gRNA	Guide ribonucleic acid
RGD	Arginine–Glycine–Aspartic motif
Angipoep-2	Peptide ligand facilitating BBB transcytosis
ABC	Accelerated blood clearance
MSCs	Mesenchymal stem cells
RVG	Neuron-targeting peptide
SPAAC	Strain-promoted azide–alkyne cycloaddition
FKBP	FK506-binding protein
FRB	FKBP–rapamycin binding domain
GMP	Good Manufacturing Practice
ISEV	International Society for Extracellular Vesicles
TGF-β	Transforming growth factor-β
